# Hierarchical organization of speech perception in human auditory cortex

**DOI:** 10.3389/fnins.2014.00406

**Published:** 2014-12-11

**Authors:** Colin Humphries, Merav Sabri, Kimberly Lewis, Einat Liebenthal

**Affiliations:** ^1^Department of Neurology, Medical College of WisconsinMilwaukee, WI, USA; ^2^Department of Psychiatry, Brigham and Women's HospitalBoston, MA, USA

**Keywords:** speech perception, auditory cortex, phonological processing, fMRI, temporal lobe, spectrotemporal cues

## Abstract

Human speech consists of a variety of articulated sounds that vary dynamically in spectral composition. We investigated the neural activity associated with the perception of two types of speech segments: (a) the period of rapid spectral transition occurring at the beginning of a stop-consonant vowel (CV) syllable and (b) the subsequent spectral steady-state period occurring during the vowel segment of the syllable. Functional magnetic resonance imaging (fMRI) was recorded while subjects listened to series of synthesized CV syllables and non-phonemic control sounds. Adaptation to specific sound features was measured by varying either the transition or steady-state periods of the synthesized sounds. Two spatially distinct brain areas in the superior temporal cortex were found that were sensitive to either the type of adaptation or the type of stimulus. In a relatively large section of the bilateral dorsal superior temporal gyrus (STG), activity varied as a function of adaptation type regardless of whether the stimuli were phonemic or non-phonemic. Immediately adjacent to this region in a more limited area of the ventral STG, increased activity was observed for phonemic trials compared to non-phonemic trials, however, no adaptation effects were found. In addition, a third area in the bilateral medial superior temporal plane showed increased activity to non-phonemic compared to phonemic sounds. The results suggest a multi-stage hierarchical stream for speech sound processing extending ventrolaterally from the superior temporal plane to the superior temporal sulcus. At successive stages in this hierarchy, neurons code for increasingly more complex spectrotemporal features. At the same time, these representations become more abstracted from the original acoustic form of the sound.

## Introduction

During the articulation of speech, vibrations of the vocal cords create discrete bands of high acoustic energy called formants that correspond to the resonant frequencies of the vocal tract. Identifying phonemic information from a speech stream depends on both the steady-state spectral content of the sound, particularly the relative frequencies of the formants, and the temporal content, corresponding to fast changes in the formants over time. Speech sounds can be divided into two general categories, vowels and consonants, depending on whether the vocal tract is open or obstructed during articulation. Because of this difference in production, vowels, and consonants have systematic differences in acoustic features. Vowels, which are produced with an open vocal tract, generally consist of sustained periods of sound with relatively little variation in frequency. Consonants, on the other hand, are voiced with an obstructed vocal tract, which tends to create abrupt changes in the formant frequencies. For this reason, vowel identification relies more heavily on the steady-state spectral features of the sound and consonant identification relies more on the momentary temporal features (Kent, 2002).

Research in animals suggests that the majority of neurons in auditory cortex encode information about both spectral and temporal properties of sounds (Nelken et al., 2003; Wang et al., 2008; Bendor et al., 2012). However, the spectrotemporal response properties of neurons vary across cortical fields. For example, in the core region of primate auditory cortex, neurons in anterior area R integrate over longer time windows than neurons in area A1 (Bendor and Wang, 2008; Scott et al., 2011), and neurons in the lateral belt have preferential tuning to sounds with wide spectral bandwidths compared to the more narrowly-tuned neurons in the core (Rauschecker et al., 1995; Rauschecker and Tian, 2004; Recanzone, 2008). This pattern of responses has been used as evidence for the existence of two orthogonal hierarchical processing streams in auditory cortex: a stream with increasing longer temporal windows extending along the posterior-anterior axis from A1 to R and a stream with increasing larger spectral bandwidth extending along the medial-lateral axis from the core to the belt (Rauschecker et al., 1995; Bendor and Wang, 2008). In addition to differences in spectrotemporal response properties within auditory cortex, other studies suggest there may also be differences between the two hemispheres, with the right hemisphere more sensitive to fine spectral details and the left hemisphere more sensitive to fast temporal changes (Zatorre et al., 2002; Poeppel, 2003; Boemio et al., 2005).

In the current study functional magnetic resonance imaging (fMRI) was used to investigate the cortical organization of phonetic feature encoding in the human brain. A main question is whether there are spatially distinct parts of auditory cortex that encode information about spectrally steady-state and dynamic sound features. Isolating feature-specific neural activity is often a problem in fMRI because different features of a stimulus may be encoded by highly overlapping sets of neurons, which could potentially result in similar patterns and levels of BOLD activation during experimental manipulations. One way to improve the sensitivity of fMRI to feature-specific encoding is to use stimulus adaptation (Grill-Spector and Malach, 2001). Adaptation paradigms rely on the fact that neural activity is reduced when a stimulus is repeated, and this effect depends on the type of information the neuron encodes. For example, a visual neuron that encodes information about spatial location might show reduced activity when multiple stimuli were presented in the same location, but would be insensitive to repetition of other features like color or shape. Adaptation-type paradigms have been used previously to study aspects of speech processing, such as phonemic categorization (Wolmetz et al., 2010), consonant (Lawyer and Corina, 2014), and vowel processing (Leff et al., 2009). In the current study, subjects listened to stimuli that were synthetic two-formant consonant-vowel (CV) syllables composed of an initial period of fast temporal change, corresponding primarily to the consonant, and a subsequent steady-state period, corresponding to the vowel. These stimuli were presented in an adaptation design, in which each trial consisted of a series of four identical syllables (e.g., /ba/, /ba/, /ba/, /ba/) followed by two stimuli that differed either in the initial transition period (e.g,. /ga/, /ga/), the steady-state period (e.g., /bi/, /bi/), or both (e.g., /gi/, /gi/). A fourth condition, in which all six stimuli were identical, was included as a baseline. The baseline condition should produce the greatest amount of stimulus adaptation and the lowest activation levels. We expected that trials with changes in the transition period compared to baseline trials would result in greater activity in neurons that encode information about fast temporal transitions, while trials with changes in the steady-state period would result in greater activity in neurons that encode information about spectral composition.

An additional question is whether any observed activation patterns represent differences in general auditory processing or differences specific to the processing of speech vowels and consonants. Previous imaging studies comparing activation during consonant and vowel processing have only used speech stimuli (Rimol et al., 2005; Obleser et al., 2010) or have used non-speech controls that were acoustically very different from speech (Joanisse and Gati, 2003), making it difficult to determine speech specificity. To address this question, we included two types of acoustically matched non-phonemic control sounds. In one type, the first formant was spectrally rotated, resulting in a sound with the same spectral complexity of speech but including a non-native (in English) formant transition. The second type of control stimuli included only one of the formants, resulting in a sound with valid English formant transitions but without harmonic spectral content. These three stimulus types (phonemic, non-phonemic, single-formant) were presented in trials of six ordered according to the four types of adaptation (steady-state change, transition change, steady-state and transition change, baseline) resulting in 12 conditions.

## Materials and methods

### Participants

FMRI data were collected from 15 subjects (8 female, 7 male; ages 21–36 years). All subjects were right-handed, native English speakers, and had normal hearing based on self report. Subjects gave informed consent under a protocol approved by the Institutional Review Board of the Medical College of Wisconsin.

### Stimuli

**Figure 1:**
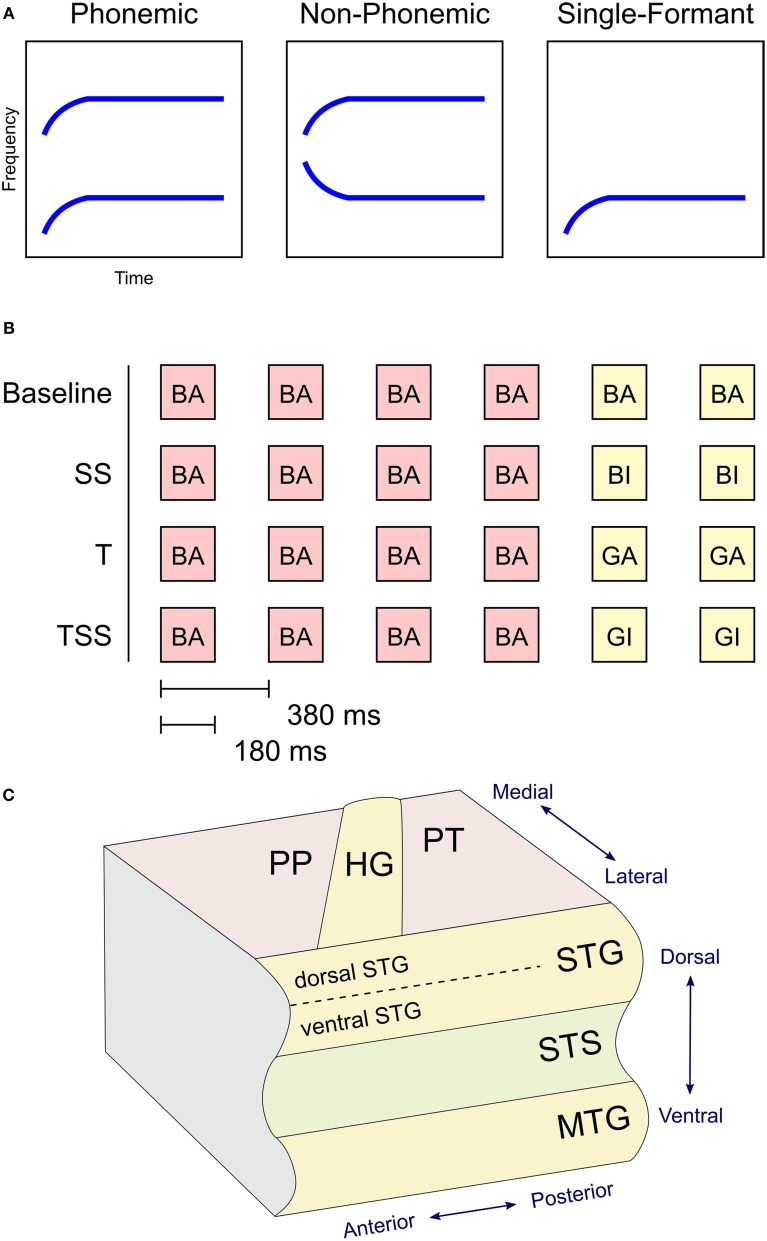
**(A)** Stimulus design. Graphs illustrate the shape of the formants used to synthesize the three types of stimuli based on the syllable /ba/. Phonemic stimuli were synthesized using the first (F1) and second (F2) formants in their canonical orientation. Non-Phonemic stimuli were composed of a standard F2 formant and a spectrally rotated F1 formant. Single-Formant stimuli only included one of the two formants (F1 or F2) from the Phonemic or Non-Phonemic stimuli. **(B)** Trial design. Examples of the four adaptation conditions are shown. Each trial consisted of six stimuli presented every 380 ms. The first four stimuli were identical. The last two stimuli varied in one of four ways. In Baseline trials the final two stimuli were identical to the first four. In Steady-State (SS) trials, the final two stimuli differed in the steady-state period (i.e., vowel). In Transient (T) trials, the final two stimuli different in the initial transition period (i.e., consonant). In the Transient and Steady-State (TSS) trials both transient and steady-state periods differed in the final two stimuli. **(C)** Diagram of superior and middle temporal cortex in the left hemisphere with labeled anatomical structures. Abbreviations: PP, Planum Polare; PT, Planum Temporale; HG, Heschl's Gyrus; STG, Superior Temporal Gyrus; STS, Superior Temporal Sulcus; MTG, Middle Temporal Gyrus.

The stimuli were synthesized speech sounds created using the KlattGrid synthesizer in Praat (http://www.fon.hum.uva.nl/praat). The acoustic parameters for the synthesizer were derived from a library of spoken CV syllables based on a male voice (Stephens and Holt, 2011). For each syllable, we first estimated the center frequencies of the first and second formants using linear predictive coding (LPC). Outliers in the formant estimates were removed. The timing of the formant estimates were adjusted so that the duration of the initial transition period of each syllable was 40 ms and the duration of the following steady-state period was 140 ms. The resulting formant time series were used as input parameters to the speech synthesizer. Three types of stimuli were generated (see Figure 1A). Phonemic stimuli were composed of both the F1 and F2 formant time courses derived from the natural syllables. Non-Phonemic stimuli were composed of the same F2 formants as the Phonemic stimuli and a spectrally rotated version of the F1 formant (inverted around the mean frequency of the steady-state period). Single-Formant stimuli contained only the F1 or F2 formant from the Phonemic and Non-Phonemic stimuli. Qualitatively, the Phonemic stimuli were perceived as English speech syllables, the Non-Phonemic stimuli were perceived as unrecognizable (non-English) speech-like sounds, and the Single-Formant stimuli were perceived as non-speech chirps (Liebenthal et al., 2005). Versions of these three types of synthesized stimuli were generated using all possible combinations of the consonants /b/, /g/, /d/, and the vowels /a/, /ae/, /i/, and /u/. Perception of the resulting stimuli was then tested in a pilot study, in which subjects (*n* = 6) were asked to identify each stimulus as one of the 12 possible CV syllables, as a different CV syllable, or as a non-speech sound. Based on the pilot study results, several of the Non-Phonemic and Single-Formant stimuli were removed from the stimulus set because they sounded too speech-like, and several of the Phonemic stimuli were removed because they were too often misidentified for another syllable or non-speech sound. A final stimulus set was chosen that consisted of Phonemic, Non-Phonemic, and Single-Formant versions of the syllables: /ba/, /bi/, /bae/, /ga/, /gi/, /gae/. In the final set, the Phonemic, Non-Phonemic, and Single-Formant stimuli were identified by participants of the pilot study as the original syllable (from which the syllable was derived and re-synthesized) at an average accuracy of 90, 46, and 13%, respectively.

The stimuli were presented using an adaptation paradigm (see Figure 1B). Each trial contained six stimuli presented every 380 ms. The first four stimuli were identical, and the final two stimuli differed from the first four in one of four ways. In the Baseline condition, the final two stimuli were identical to the first four. In the Steady-State (SS) condition, the final two stimuli differed from the first four in the steady-state vowel (e.g., /ba/, /ba/, /ba/, /ba/, /bi/, /bi/). In the Transition (T) condition, the final stimuli differed in their transition period (e.g., /ba/, /ba/, /ba/, /ba/, /ga/, /ga/). In the Transition Steady-State (TSS) condition, both the steady-state and transition periods differed in the final stimuli (e.g., /ba/, /ba/, /ba/, /ba/, /gi/, /gi/).

### Procedure

Each participant was scanned in two sessions occurring on different days. Each scanning session consisted of a high resolution anatomical scan (SPGR sequence, axial orientation, 180 slices, 256 × 240 matrix, FOV = 240 mm, 0.9375 × 1.0 mm^2^ resolution, 1.0 mm slice thickness) and five functional scans (EPI sequence, 96 × 96 matrix, FOV = 240 mm, 2.5 × 2.5 mm^2^ resolution, 3 mm slice thickness, *TA* = 1.8 s, *TR* = 7.0 s). Functional scans were collected using a sparse-sampling procedure in which stimuli were presented during a silent period between MR image collection (Hall et al., 1999).

The experiment was organized in a 3 × 4 factorial design with the three stimulus types (Phonemic, Non-Phonemic, and Single-Formant) presented in four different adaptation configurations (TSS, T, SS, and Control) resulting in a total of 12 conditions. The conditions were presented in trials consisting of six stimuli presented every 380 ms followed by a single MR volume acquisition lasting 1.8 s. A small percentage (*p* = 0.1) of trials were missing either one or two of the six stimuli. To ensure that subjects were attending to the stimuli during the experiment, subjects were required to hit a button when they detected a missing stimulus. Compliance with the task was assessed, but image data from the trials with missing stimuli were excluded from the analysis. Within each run 8 trials were presented per condition producing a total of 80 trials per condition across both sessions. An additional 8 trials of rest (i.e., no stimulus) were included in each run. Trials were presented in blocks containing 4 trials of the same condition. The order of the blocks was randomized across runs and across participants.

Sounds were presented binaurally with in-ear electrostatic headphones (Stax SR-003; Stax Ltd, Saitama, Japan). Additional protective ear muffs were placed over the headphones to attenuate scanner noise.

The fMRI data were analyzed using AFNI (Saad et al., 2009). Initial preprocessing steps included motion correction and co-registration between the functional and anatomical scans. The anatomical volumes from each subject were aligned using non-linear deformation to create a study-specific atlas using the program ANTS (Avants and Gee, 2004). The functional data were resampled (voxel size = 2.5 × 2.5 × 2.5 mm^3^) into the atlas space and spatially filtered using a Gaussian window (FWHM = 5 mm). Our primary research questions were focused on differences in activation in auditory areas, therefore, we confined our analysis to a set of voxels that included the entire superior, middle, and inferior temporal lobe and extending into the inferior parietal and lateral occipital lobes.

Estimates of the activation levels for the 12 conditions were calculated using the AFNI command 3dREMLfit, which models the data using a generalized least squares analysis with a restricted maximum likelihood (REML) estimate of temporal auto-correlation. Contrasts between conditions were evaluated at the group level using a mixed-effects model. To correct for increased type 1 error due to multiple comparisons, the voxels in the resulting statistical maps were initially thresholded at *p* < 0.01, grouped into contiguous clusters, and then thresholded at *p* < 0.05 using a cluster-size threshold of 29 determined using the AFNI command 3dClustStim. An additional analysis using an initial threshold of *p* < 0.05 and a cluster-size threshold of 108 voxels (*p* < 0.05, corrected) was performed on one of the contrasts. Mean effect sizes for each cluster were calculated by dividing the amplitude of the contrast values by the mean signal level and then taking a mean across all the voxels in the cluster. The maps are displayed on an inflated surface brain of the ANTS-derived atlas created using Freesurfer (Dale et al., 1999). A diagram of the location of the anatomical labels used to describe the results is displayed in Figure 1C.

## Results

**Figure 2:**
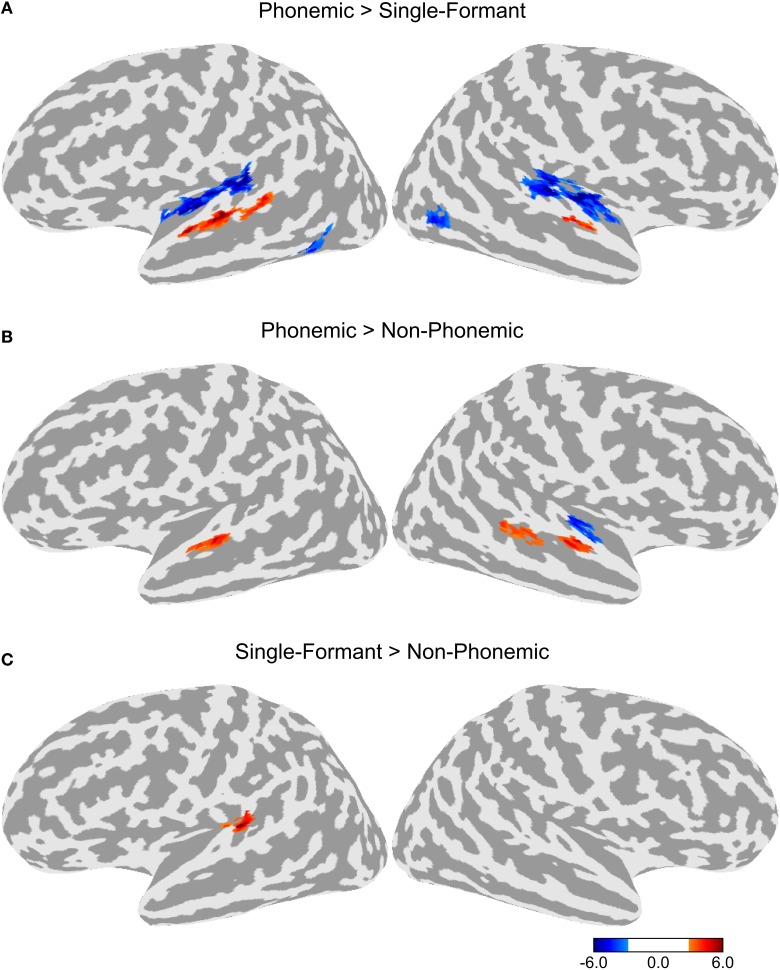
**Differences in activation between the three stimulus types collapsed across the four adaptation conditions**. **(A)** Comparison of the activation levels in the Phonemic and Single-Formant trials. **(B)** Comparison between the Phonemic and Non-Phonemic trials. **(C)** Comparison between the Single-Formant and Non-Phonemic trials.

Differences in BOLD activation between the three stimulus types are shown in Figure 2. Each contrast represents the difference in activation between two of the three stimulus types collapsed across the four adaptation conditions. Greater levels of activity were observed during Phonemic trials compared to either the Non-Phonemic or Single-Formant trials in the superior temporal gyrus (STG), bilaterally. More specifically, the voxels in this activation cluster were located on the more inferior side of the curve of the STG (see **Figure 4**), which we refer to as ventral STG, and distinguish this area from the more superior side of the STG, which we refer to as dorsal STG. There was less activity during Phonemic trials compared to Single-Formant trials in both hemispheres in the superior temporal plane (STP), specifically the medial portion, and in the posterior part of the middle temporal sulcus. Less activity during Phonemic compared to Non-Phonemic trials was found in a smaller cluster in the planum polare in the right hemisphere. Single-Formant trials had greater activity than Non-Phonemic trials in the left planum temporale.

**Figure 3:**
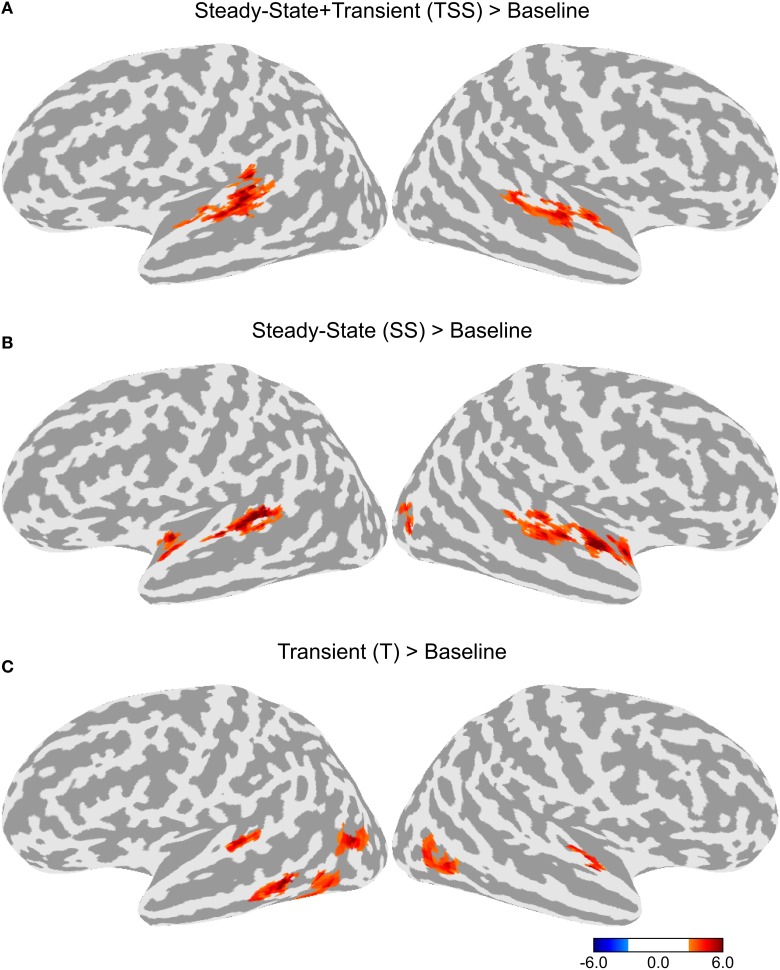
**Differences in activation between each adaptation condition and Baseline collapsed across stimulus type**. **(A)** Contrast between activation levels in the Transient and Steady-State (TSS) condition against the Baseline condition. **(B)** Contrast between the Steady-State (SS) condition and the Baseline condition. **(C)** Contrast between the Transient (T) condition and the Baseline condition.

To test for adaptation effects, each of the three adaptation conditions (T, SS, and TSS) were compared to the Baseline adaptation condition, in which all six stimuli in the trial were identical. Each of the adaptation contrasts included all three stimulus types. The resulting maps are shown in Figure 3. All three adaptation conditions demonstrated greater activity than the Baseline condition in the dorsal STG, bilaterally. The comparison of SS against Baseline produced a cluster of activation extending along the dorsal STG both anterior and posterior to Heschl's gyrus (HG). The TSS condition activated a similar set of areas. The T condition appeared to have the smallest extent of activation confined to a section of cortex along the middle of the STG. Additional adaptation effects were observed outside of auditory cortex. Significant clusters of activation for the T condition were observed in the left middle temporal gyrus (MTG) and bilateral middle temporal sulcus. In addition, activation for the SS was found in the right lateral occipital sulcus.

**Figure 4:**
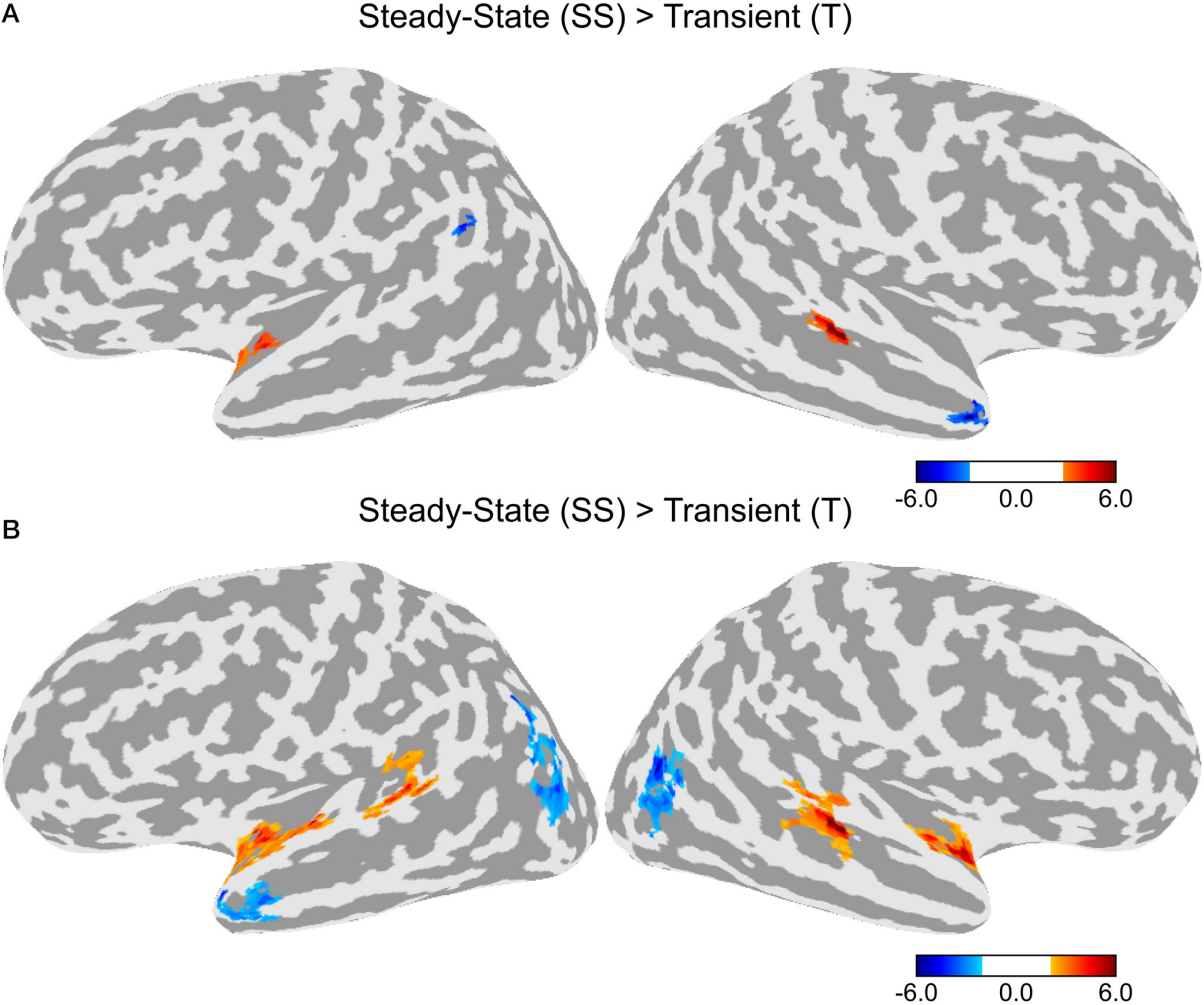
**Differences in activation between the Transient (T) and Steady-State (SS) adaptation conditions**. **(A)** Contrast between T and SS using an initial threshold of *p* < 0.01 (α = 0.05, corrected). **(B)** Contrast between T and SS using an initial threshold of *p* < 0.05 (α = 0.05, corrected).

A direct contrast between the T and SS conditions is shown in Figure 4A. Greater activity in the SS condition was observed in a cluster in the left anterior STG and another cluster in the right posterior STG. Greater activity in the T condition was observed in the left superior marginal gyrus and the right temporal pole. Given that differences in activation levels between the two types of adaptation could be small resulting in a lower statistical effect, we ran an additional contrast using a lower initial threshold of *p* < 0.05 with the same corrected alpha level of 0.05 (see Figure 4B). In this contrast, there was greater activity in the SS condition in bilateral anterior STG and bilateral posterior STG. There was no difference between T and SS in the middle section of the STG just lateral to HG. Greater activation for the T condition was observed in bilateral lateral occipital complex and the left temporal pole.

**Figure 5:**
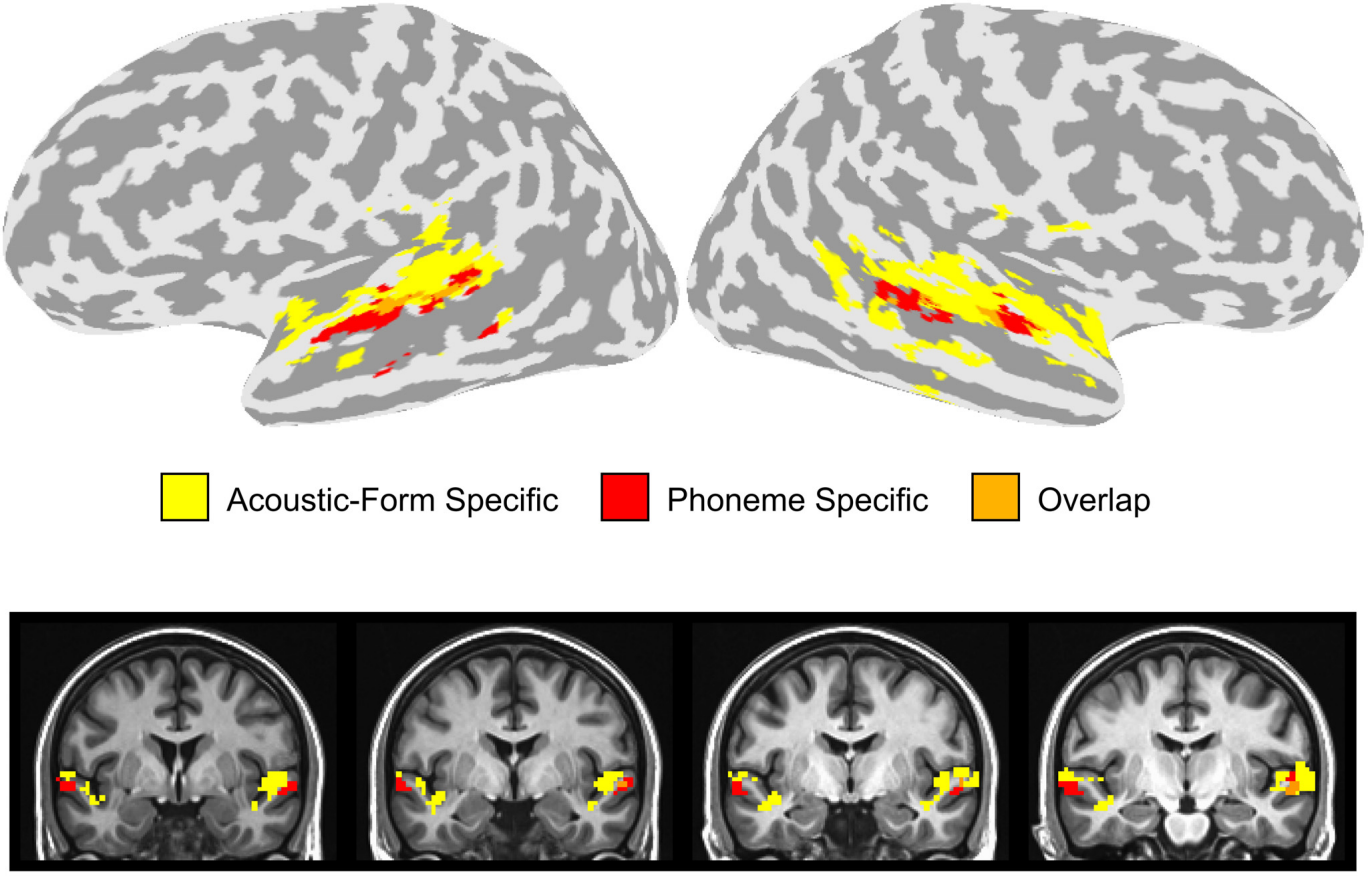
**Overlay of stimulus and adaptation effects in the STG**. Voxels in the dorsal or ventral STG that were significantly active in the Phonemic > Single-Formant or Phonemic > Non-Phonemic contrasts (i.e., phoneme specific) are displayed in red. Voxels in the STG that were significant in any of the **three** adaptation contrasts (i.e., acoustic-form specific) are shown in yellow. Overlapping voxels are colored orange.

In order to compare the location of the activation clusters identified in the dorsal and ventral STG, we overlaid the activation maps for the combination of the two stimulus contrasts (Phonemic > Non-Phonemic and Phonemic > Single-Formant) and the three adaptation contrasts (SS > Baseline, T > Baseline, and TSS > Baseline) (Figure 5). Voxels that were significant for either of the two stimulus contrasts are displayed in red, voxels significant for any of the three adaptation contrasts are in yellow, and overlapping voxels are in orange. Activation clusters showing preferential response to phonemic stimuli were ventral and adjacent to clusters showing adaptation effects related to changes in acoustic form with little overlap between the clusters.

In the sections of cortex in the dorsal and ventral STG that showed activation in the stimulus and adaptation contrasts, we did not find significant interactions between adaptation and stimulus type. However, significant interaction effects were seen in several clusters outside of this region (see Table 1). The interaction between SS and Single-Formant over Phonemic showed a cluster in the right inferior parietal lobe and between SS and Single-Formant over Non-Phonemic in the left middle temporal sulcus. The interaction between T and Phonemic over Single-Formant was seen in the left anterior STS. The interaction between TSS and Phonemic over Non-Phonemic showed activation in the right posterior STS/STG and between TSS and Single-Formant over Non-Phonemic in the bilateral posterior STG and bilateral MTG.

**Table 1:** **FMRI Activation Clusters**.

**Hemi**	**Center**			**Peak**			***t*-value**	**Cluster size (voxels)**	**Mean effect size (%)**	**Region**
	***X***	***Y***	***Z***	***X***	***Y***	***Z***				
**PHONEMIC > NON-PHONEMIC**										
L	−60.5	−11.0	−3.6	−62.9	−12.9	−4.3	5.19	57	0.23	superior temporal gyrus
R	48.3	−29.9	−0.4	45.4	−37.7	5.4	4.02	33	0.15	superior temporal gyrus
R	59.5	−4.2	−6.8	58.3	−9.5	−9.2	4.81	34	0.18	superior temporal gyrus
**NON-PHONEMIC > PHONEMIC**										
R	48.4	−8.3	0.3	53.0	−1.9	4.7	4.88	69	0.16	planum polare (medial)
**PHONEMIC > SINGLE-FORMANT**										
L	−61.4	−17.9	−1.1	−62.9	−12.9	−4.3	7.27	190	0.27	ventral superior temporal gyrus
R	60.1	−0.9	−5.6	61.0	−2.7	−1.4	4.86	32	0.20	ventral superior temporal gyrus
**SINGLE-FORMANT > PHONEMIC**										
L	−42.6	−60.6	−6.4	−43.1	−66.7	2.5	4.85	42	0.08	inferior temporal sulcus
L	−39.7	−23.6	5.3	−40.9	−28.6	16.8	7.94	185	0.16	planum polare/temporale (medial)
R	43.4	−60.3	–1.1	45.7	−57.8	−0.0	4.45	47	0.10	inferior temporal sulcus
R	44.6	−19.4	7.6	56.0	−23.7	5.7	7.03	285	0.17	planum polare/temporale (medial)
**SINGLE-FORMANT > NON-PHONEMIC**										
L	−38.7	−32.9	13.8	−38.2	−32.2	11.3	6.49	59	0.11	planum temporale (medial)
**STEADY-STATE ADAPTATION > BASELINE**										
L	−62.3	−27.1	7.5	−65.1	−35.2	12.1	8.71	220	0.15	superior temporal gyrus (posterior)
L	−46.1	−2.6	−13.1	−41.4	−8.3	−11.5	5.54	60	0.15	superior temporal gyrus (anteior)
R	36.7	−82.1	11.1	38.0	−84.2	8.1	4.82	33	0.08	lateral occipital gyrus
R	55.5	−15.2	−3.1	52.8	2.1	−5.2	10.03	564	0.17	superior temporal gyrus
**TRANSIENT ADAPTATION > BASELINE**										
L	−59.6	−29.3	6.6	−59.9	−22.2	6.6	5.28	64	0.10	superior temporal gyrus
L	−58.5	−39.6	−9.2	−59.7	−43.7	−7.8	6.81	125	0.11	middle temporal gyrus
L	−41.8	−60.2	−10.5	−35.2	−65.3	−7.0	6.49	158	0.12	lateral occipital gyrus
L	−37.7	−72.4	13.0	−37.6	−70.6	12.4	5.49	43	0.09	inferior temporal sulcus
R	41.6	−66.7	4.4	38.0	−78.8	7.1	5.06	154	0.10	inferior temporal sulcus
R	54.9	2.1	−4.2	60.8	7.5	−6.3	5.47	37	0.14	superior temporal gyrus
**STEADY-STATE AND TRANSIENT ADAPTATION > BASELINE**										
L	−57.2	−24.2	6.7	−49.1	−27.9	4.5	7.71	286	0.14	superior temporal gyrus
R	58.8	−17.8	0.5	50.2	−2.8	−1.2	5.57	296	0.15	superior temporal gyrus
**(STEADY-STATE ADAPTATION > BASELINE) X (SINGLE-FORMANT > PHONEMIC)**										
R	41.2	−51.7	58.7	51.5	−52.0	53.9	28.79	36	0.56	inferior parietal sulcus
**(STEADY-STATE ADAPTATION > BASELINE) X (SINGLE-FORMANT > NON-PHONEMIC)**										
L	−57.0	−57.6	−21.8	−57.0	−56.7	−20.6	5.24	45	0.27	inferior temporal sulcus
**(TRANSIENT ADAPTATION > BASELINE) X (PHONEMIC > SINGLE-FORMANT)**										
L	−52.7	−2.6	−15.0	−49.6	−6.6	−17.9	6.18	47	0.09	superior temporal sulcus (anterior)
**(STEADY-STATE AND TRANSIENT ADAPTATION > BASELINE) X (PHONEMIC > NON-PHONEMIC)**										
R	56.7	−44.3	20.3	51.0	−44.3	15.7	5.68	56	0.08	superior temporal gyrus (posterior)
R	63.8	−49.5	5.4	64.3	−43.3	3.2	4.24	40	0.13	superior temporal sulcus (posterior)
**(STEADY-STATE AND TRANSIENT ADAPTATION > BASELINE) X (SINGLE-FORMANT > NON-PHONEMIC)**										
L	−59.1	−55.1	−2.3	−56.7	−62.0	−1.3	5.19	189	0.12	inferior temporal sulcus
L	−57.7	−29.1	13.5	−54.2	−34.1	17.9	5.75	41	0.08	superior temporal gyrus (posterior)
R	59.7	−34.4	6.6	56.2	−34.4	7.7	6.40	74	0.08	superior temporal gyrus (posterior)
R	60.4	−20.5	−11.4	63.9	−20.6	−10.3	5.55	33	0.12	middle temporal gyrus
**TRANSIENT ADAPTATION > STEADY-STATE ADAPTATION**										
L	−51.3	−50.7	38.1	−53.8	−53.2	36.8	5.55	31	0.11	super marginal gyrus
R	44.6	14.1	−37.2	47.0	13.9	−34.9	6.00	32	0.24	temporal pole
**STEADY-STATE ADAPTATION > TRANSIENT ADAPTATION**										
L	−45.4	−4.1	−6.7	−46.7	−6.0	3.4	7.15	40	0.15	superior temporal gyrus (anterior)
R	55.9	−22.3	0.8	53.2	−19.3	−1.2	9.72	52	0.12	superior temporal gyrus (posterior)
**TRANSIENT ADAPTATION > STEADY-STATE ADAPTATION^a^**										
L	−45.6	7.9	−33.8	−47.4	22.4	−26.4	4.60	113	0.16	temporal pole
L	−42.0	−66.7	27.7	−53.8	−53.2	36.8	5.55	380	0.10	lateral occipital gyrus
R	44.7	−69.0	13.8	40.8	−72.9	27.3	6.31	217	0.08	lateral occipital gyrus
**STEADY-STATE ADAPTATION > TRANSIENT ADAPTATION^a^**										
L	−62.0	−34.8	11.5	−70.6	−27.2	10.7	5.01	117	0.12	superior temporal gyrus (posterior)
L	−45.8	−0.8	−7.9	−46.7	−6.0	3.4	7.15	151	0.12	superior temporal gyrus (anterior)
R	47.6	2.9	−11.2	50.0	10.5	−3.7	5.63	111	0.13	superior temporal gyrus (anterior)
R	58.6	−25.3	2.9	53.2	−19.3	−1.2	9.72	209	0.10	superior temporal gyrus (posterior)

Unless noted, all contrasts are threshold = *p* < 0.01 (0.05 corrected).

a threshold = *p* < 0.05 (0.05 corrected).

## Discussion

We investigated the patterns of neural activity associated with perception of the transition and steady-state portions of CV syllables and non-speech controls using fMRI. Two adjacent but distinct regions in the superior temporal lobe were identified that were affected by manipulations of either feature-specific adaptation or stimulus type (Figure 5). On the dorsal side of the STG extending into the STP, voxels had reduced activity during the repetition of both the transition and steady-state portions of the sound regardless of whether the stimulus was Phonemic, Non-Phonemic, or Single-Formant. On the ventral side of the STG extending into the STS, voxels displayed higher levels of activity during Phonemic compared to Non-Phonemic and Single-Formant trials but were not sensitive to adaptation of acoustic features. Brain areas showing selectivity to acoustic form (i.e., to the adaptation condition) and brain areas showing selectivity to phonemes were located adjacent to each other in the dorsal and ventral STG, with little overlap between them. Finally in bilateral STP, increased activity was observed for the Non-Phonemic and Single-Formant sounds over the Phonemic sounds.

Adaptation effects due to stimulus repetition were observed in the bilateral dorsal STG extending into the STP. This region has been identified in a wide range of studies looking at auditory and speech processing (Alho et al., 2014), and it appears to play a role in processing stimuli with “complex” spectrotemporal structure. For example, higher levels of activity in the bilateral dorsal STG are observed for sounds with multiple spectral components (Schönwiesner et al., 2005; Lewis et al., 2012; Moerel et al., 2013; Norman-Haignere et al., 2013) or sounds containing temporal modulations (Schönwiesner et al., 2005; Herdener et al., 2013; Santoro et al., 2014) compared to simple auditory controls like tones or noise. Greater activity is also observed in this area for stimuli with more complex spectrotemporal structure, such as speech, animal vocalizations, or environmental sounds (Altmann et al., 2007; Joly et al., 2012; Lewis et al., 2012). In the current study, the bilateral dorsal STG demonstrated adaptation to the transition and steady-state portions of the stimulus regardless of whether the stimulus was phonemic or not, suggesting that it plays a role in representing certain types of spectrotemporal features that are relevant (but not exclusive) to phoneme perception, such as the multi-frequency harmonics that form the steady-state period or the rapid frequency sweeps that occur during the transition period of speech syllables.

Increased activity in the dorsal STG was observed for all three adaptation conditions compared to baseline, however, there were some differences in the patterns of activation. First, the activation clusters in the two conditions with a change in the steady-state period (SS and TSS) were larger than those for the transition condition (T). Second, direct contrasts between the T and SS conditions (Figure 4) showed greater activity for SS in bilateral anterior and posterior STG, suggesting that neurons encoding information about the steady-state period are located across the entire STG, while the transition period is primarily encoded by neurons in an areas confined to the middle STG lateral to HG. The steady-state and transition periods of the stimuli used in the experiment have different types of spectrotemporal structure. The transition period consists of relatively fast changes in spectral content, while the steady-state period has relatively little spectral variation over time. It is possible that neural processing during these two time periods involves different populations of neurons, which are sensitive to different types of spectrotemporal features. Studies in monkeys suggest that neurons in more anterior cortical fields (R and AL) have longer latencies and longer sustained responses than the more centrally-located A1, suggesting that these neurons process acoustic information over longer time windows (Tian and Rauschecker, 2004; Bendor and Wang, 2008; Scott et al., 2011). If the anterior auditory neurons in human have similar windows of integration as in the monkey (>100 ms), then these neurons would be less sensitive to the fast temporal changes during the transition period, resulting in less adaptation in the T condition. It has been suggested that these anterior auditory fields form an auditory ventral stream, in which both acoustic and linguistic information is processed at increasing longer time scales (Rauschecker and Scott, 2009). In speech, much of the longer acoustic information (i.e., prosody) is derived by tracking pitch intonation, which is primarily determined from the vowel steady-state periods. Although these neurons might be less sensitive to fast temporal changes during the transition period, they might be optimally tuned to detecting changes in the steady-state period. In line with this view, is the finding that sentences with scrambled prosody show reduced activation compared to normally spoken sentences in bilateral anterior STG (Humphries et al., 2005). In addition to the anterior STG, the current study also found a similar activation pattern in the posterior STG. This set of areas is thought to be part of a dorsal auditory stream involved in sound localization and speech-motor coordination (Hickok and Poeppel, 2007; Rauschecker and Scott, 2009; Liebenthal et al., 2013). Like the anterior areas, decreased sensitivity in the posterior STG to the transition period could be related to longer processing windows. In contrast, the finding of high activity levels for both the T and SS conditions in a section of the middle STG, adjacent to the ventral STG area that showed greater response to the Phonemic condition, suggests that these two types of acoustic features are important for phoneme processing.

Greater activation for the T condition was found in several areas outside of auditory cortex. It has been suggested that vowels and consonants contribute differently to speech perception, with vowels containing the majority of acoustic information about prosody and segmentation, and consonants providing linguistic-based information about lexical identity (Nespor et al., 2003). The activation differences between T and SS could also be related to this distinction. Greater sensitivity to the steady-state periods corresponding to vowels was found in purely auditory regions and greater sensitivity to the transition period corresponding to the consonant was found in parts of the cortex considered to be heteromodal and possibly involved lexical semantic processing.

Higher levels of activity in the bilateral ventral STG were seen for the Phonemic condition compared to the Non-Phonemic and Single-Formant sounds. This is consistent with findings from a large body of studies that have found greater activation in this area in response to speech syllables compared to non-speech auditory controls (Obleser et al., 2007; Leaver and Rauschecker, 2010; Liebenthal et al., 2010, 2005; Leech and Saygin, 2011; Woods et al., 2011). Furthermore, the left ventral STG has been shown to have categorical response to speech syllables varied along an acoustic continuum suggesting that this area is involved in abstract representations of sound (Liebenthal et al., 2005; Joanisse et al., 2007). In the current study, the Non-Phonemic and Single-Formant stimuli were synthesized with parameters very closely matching the spectrotemporal composition of the Phonemic stimuli. Thus, the observed differences in activation cannot be attributed simply to differences in acoustic form. The fact that this area did not respond to adaptation further supports the view that it encodes abstract representations of sound.

The results from the current study support the view that there are multiple hierarchical processing streams extending from primary auditory cortex to anterior, posterior, and lateral parts of the temporal lobe (Rauschecker et al., 1995; Kaas and Hackett, 2000; Hickok and Poeppel, 2007; Rauschecker and Scott, 2009). The dorsal and ventral parts of the STG observed in the current study represent two stages along these hierarchical pathways. Neurons in the dorsal STG encode information about complex spectrotemporal features by integrating across simpler acoustic features represented in earlier stages in the hierarchy in primary auditory cortex. The ventral STG, in turn, integrates information from the dorsal STG to build more complex representations related specifically to phonemic patterns. As the representations become more complex, they also become more abstract with reduced sensitivity to acoustic form, allowing categorical identification of acoustically varying sounds, such as speech phonemes. In addition to this dorsal/ventral hierarchy, the difference observed here between adaptation to the transition and steady-state segments of the stimuli suggests that there are important anterior-posterior differences in the superior temporal cortex beyond those associated with the dual-stream model of auditory processing. The results are consistent with the existence of several functional pathways tuned to different types of acoustic information, specifically only slow spectrally changing information in anterior and posterior STG and both slow and fast spectral information in the middle STG.

Finally, on the medial side of the STP, a larger response was found for Non-Phonemic and Single-Formant sounds compared to Phonemic sounds. This area did not activate in the adaptation contrasts. Other studies have observed a similar preference for non-speech over speech sounds in this region (Tremblay et al., 2013). Its location in medial auditory cortex suggests that it is homologous to the medial belt identified in the monkey. Interestingly, a study of the response properties of medial belt neurons in the monkey suggests a similar preference for spectral wide-band stimuli as in lateral belt neurons (Kuśmierek and Rauschecker, 2009). However, unlike lateral belt neurons, medial belt neurons do not show preferential responses to monkey vocalizations (Kuśmierek and Rauschecker, 2009). Thus, it is possible that the preference for non-phonemic sounds in medial auditory cortex could represent a tuning to sounds with unfamiliar, simpler harmonic structure.

In conclusion, we identified distinct regions of auditory cortex that were differentially sensitive to acoustic form and stimulus type, suggesting a hierarchical organization of auditory fields extending ventrolaterally from primary auditory cortex to the STS and with varying sensitivity to acoustic form along the anterior to posterior axis of the STG. These results extend our understanding of the brain areas involved in auditory object identification and speech perception.

### Conflict of interest statement

The authors declare that the research was conducted in the absence of any commercial or financial relationships that could be construed as a potential conflict of interest.
